# Complete genome sequence of *Treponema succinifaciens* type strain (6091^T^)

**DOI:** 10.4056/sigs.1984594

**Published:** 2011-06-30

**Authors:** Cliff Han, Sabine Gronow, Hazuki Teshima, Alla Lapidus, Matt Nolan, Susan Lucas, Nancy Hammon, Shweta Deshpande, Jan-Fang Cheng, Ahmed Zeytun, Roxanne Tapia, Lynne Goodwin, Sam Pitluck, Konstantinos Liolios, Ioanna Pagani, Natalia Ivanova, Konstantinos Mavromatis, Natalia Mikhailova, Marcel Huntemann, Amrita Pati, Amy Chen, Krishna Palaniappan, Miriam Land, Loren Hauser, Evelyne-Marie Brambilla, Manfred Rohde, Markus Göker, Tanja Woyke, James Bristow, Jonathan A. Eisen, Victor Markowitz, Philip Hugenholtz, Nikos C. Kyrpides, Hans-Peter Klenk, John C. Detter

**Affiliations:** 1 DOE Joint Genome Institute, Walnut Creek, California, USA; 2 Los Alamos National Laboratory, Bioscience Division, Los Alamos, New Mexico, USA; 3 DSMZ - German Collection of Microorganisms and Cell Cultures GmbH, Braunschweig, Germany; 4 Biological Data Management and Technology Center, Lawrence Berkeley National Laboratory, Berkeley, California, USA; 5 Oak Ridge National Laboratory, Oak Ridge, Tennessee, USA; 6 HZI – Helmholtz Centre for Infection Research, Braunschweig, Germany; 7 University of California Davis Genome Center, Davis, California, USA; 8 Australian Centre for Ecogenomics, School of Chemistry and Molecular Biosciences, The University of Queensland, Brisbane, Australia

**Keywords:** anaerobic, motile, Gram-negative, mesophilic, chemoorganotrophic, *Spirochaetaceae*, GEBA

## Abstract

*Treponema succinifaciens* Cwyk and Canale-Parola 1981 is of interest because this strictly anaerobic, apathogenic member of the genus *Treponema* oxidizes carbohydrates and couples the Embden-Meyerhof pathway *via* activity of a pyruvate-formate lyase to the production of acetyl-coenzyme A and formate. This feature separates this species from most other anaerobic spirochetes. The genome of *T. succinifaciens* 6091^T^ is only the second completed and published type strain genome from the genus *Treponema* in the family *Spirochaetaceae*. The 2,897,425 bp long genome with one plasmid harbors 2,723 protein-coding and 63 RNA genes and is a part of the *** G****enomic* *** E****ncyclopedia of* *** B****acteria and* *** A****rchaea * project.

## Introduction

Strain 6091^T^ (= DSM 2489 = ATCC 33096 = JCM 13475) is the type strain of *Treponema succinifaciens* [1,2]. Currently, there are 25 species placed in the genus *Treponema* [3]. The species epithet is derived from the Latin noun *acidum succinicum* meaning *succinic acid* and the Latin verb *facio* meaning *to make, produce*, referring to the succinic acid-producing property of the species [1]. *T. succinifaciens* was isolated from the colon of swine, and first described as *small spirochete* by Harris *et al.* in 1972 [4]. In 1974 it was published that strain 6091^T^ belonged to a group of harmless inhabitants of the intestine of healthy pigs and had no pathogenic potential [5]. No further isolates have been described and strain 6091^T^ was designated the type strain of the new species *T. succinifaciens* in 1979 [1]. Here we present a summary classification and a set of features for *T. succinifaciens* 6091^T^, together with the description of the complete genomic sequencing and annotation.

## Classification and features

A representative genomic 16S rRNA sequence of *T. succinifaciens* was compared using NCBI BLAST [6] under default settings (e.g., considering only the high-scoring segment pairs (HSPs) from the best 250 hits) with the most recent release of the Greengenes database [7] and the relative frequencies of taxa and keywords (reduced to their stem [8]) were determined, weighted by BLAST scores. The most frequently occurring genera were *Treponema* (97.5%) and *Spirochaeta* (2.5%) (32 hits in total). Regarding the single hit to sequences from members of the species, the average identity within HSPs was 97.7%, whereas the average coverage by HSPs was 96.1%. Regarding the 14 hits to sequences from other members of the genus, the average identity within HSPs was 89.2%, whereas the average coverage by HSPs was 54.1%. Among all other species, the one yielding the highest score was *Treponema socranskii* (AY369254), which corresponded to an identity of 89.8% and an HSP coverage of 55.7%. (Note that the Greengenes database uses the INSDC (= EMBL/NCBI/DDBJ) annotation, which is not an authoritative source for nomenclature or classification.) The highest-scoring environmental sequence was EU462604 ('Evolution mammals and their gut microbes Sumatran orangutan feces clone orang2 aai66a03'), which showed an identity of 99.6% and an HSP coverage of 91.6%. The most frequently occurring keywords within the labels of environmental samples which yielded hits were 'gut' (11.2%), 'evolut, fece, mammal, microb' (8.2%), 'baboon, hamadrya' (6.3%), 'termit' (5.1%) and 'homogen' (2.2%) (218 hits in total). The most frequently occurring keywords within the labels of environmental samples which yielded hits of a higher score than the highest scoring species were 'gut' (12.1%), 'evolut, fece, mammal, microb' (11.5%), 'baboon, hamadrya' (9.9%), 'rumen' (1.3%) and 'termit' (1.1%) (77 hits in total). These keywords fit to the ecological and physiological properties reported for strain 6091^T^ in the original description [1].

**Figure 1:**
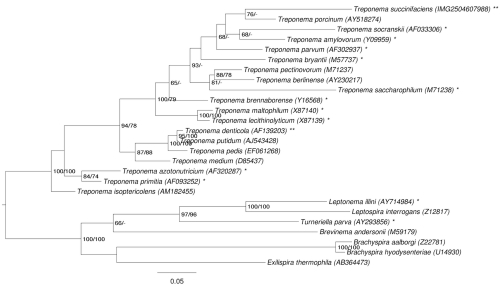
Phylogenetic tree highlighting the position of *T. succinifaciens* relative to the type strains of the other species within the phylum '*Spirochaetes*'. The tree was inferred from 1,350 aligned characters [9,10] of the 16S rRNA gene sequence under the maximum likelihood (ML) criterion [11]. Rooting was done initially using the midpoint method [12] and then checked for its agreement with the current classification (Table 1). The branches are scaled in terms of the expected number of substitutions per site. Numbers adjacent to the branches are support values from 1,000 ML bootstrap replicates [13] (left) and from 1,000 maximum parsimony bootstrap replicates [14] (right) if larger than 60%. Lineages with type strain genome sequencing projects registered in GOLD [15] are marked with one asterisk, those also listed as 'Complete and Published' (as well as the target genome) with two asterisks [16].

Figure 1 shows the phylogenetic neighborhood of *T. succinifaciens* in a 16S rRNA based tree. The sequences of the four 16S rRNA gene copies in the genome differ from each other by up to seven nucleotides, and differ by up to 14 nucleotides from the previously published 16S rRNA sequence (M57738), which contains 26 ambiguous base calls.

**Figure 2:**
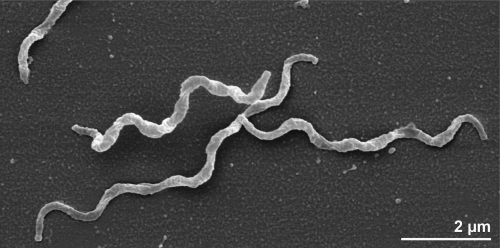
Scanning electron micrograph of *T. succinifaciens* 6091^T^

The cells of *T. succinifaciens* are of helical shape (0.3 × 4-8 µm) and usually exhibit irregular coiling (Figure 2). Cells are up to 16 µm long and also chains of cells may occur in culture [1]. *T. succinifaciens* is a Gram-negative and non spore-forming bacterium (Table 1). The organism displays temperature-dependent motility of translational, rotary and flexing movements; at 23–25°C no translational movement can be observed and rotation is slow, whereas at 37°C cells are very mobile (average velocity: 15µm/s) [1]. *T. succinifaciens* harbors two periplasmic fibrils inserted near each end of the cell [1]. The genome of *T. succinifaciens* contains 63 genes involved in motility (see below). The organism is a strictly anaerobic chemoorganotroph [1]. *T. succinifaciens* requires rumen fluid in media for good growth, replacement with a mixture of short-chain fatty acids leads to reduced growth yields [1]. The temperature range for growth is between 22°C and 43°C, with an optimum between 35°C and 39°C [1]. The organism is catalase-negative and does not grow in the presence of 6.5% NaCl [1]. *T. succinifaciens* requires CO_2_ for growth and is able to utilize arabinose, xylose, glucose, mannose, galactose, maltose, lactose, cellobiose, dextrin and starch for fermentation. Sugar alcohols, amino acids and other organic acids cannot be fermented by the organism [1]. Major fermentation products of glucose are acetate, formate, succinate and lactate, whereas pyruvate, acetoin and 2,3-butanediol are formed in minor amounts [1]. Assays of enzymatic activities showed that *T. succinifaciens* dissimilates glucose *via* the Embden-Meyerhof pathway [1]. It was shown that pyruvate is metabolized through the activity of pyruvate formate lyase to yield acetyl-coenzyme A and formate, which is in contrast to other spirochetes that degrade pyruvate to acetyl-coenzyme A, CO_2_ and H_2_ [1]. Furthermore, *T. succinifaciens* is capable of CO_2_ fixation for the production of succinate [1]. Also, the organism possesses enzymatic activity of adenine deaminase, phosphoribosyltransferase (for adenine, guanine and hypoxanthine), nucleotidase (for AMP, IMP and GMP), nucleoside phosphorylase (for adenosine, guanosine and inosine) and nucleoside hydrolase (for inosine and guanosine) [28]. Whether these activities are important for the survival of *T. succinifaciens* under nutrient deprivation or for adaptation to environmental stress is still unclear. An outer membrane-associated serine protease, which was found in several pathogenic spirochetes and also in *T. succinifaciens*, might be involved in the survival within the intestine, however, a role in pathogenesis can so far not be excluded [29]. *T. succinifaciens* is susceptible to penicillin G (4 units/ml), cephalotin (4 µg/ml) and chloramphenicol (4 µg/ml). Growth of the organism is not impaired by erythromycin (4 µg/ml), oxytetracycline (4 µg/ml), polymyxin B (40 units/ml), rifampin (4 µg/ml), streptomycin (4 µg/ml), tetracycline (4 µg/ml) or vancomycin (4 µg/ml) [1].

**Table 1:** Classification and general features of *T. succinifaciens* 6091^T^ according to the MIGS recommendations [17] and the NamesforLife database [3].

**MIGS ID**	**Property**	**Term**	**Evidence code**
	Current classification	Domain *Bacteria*	TAS [18]
		Phylum “*Spirochaetae*”	TAS [19]
		Class “*Spirochaetes*”	TAS [20]
		Order *Spirochaetales*	TAS [21,22]
		Family *Spirochaetaceae*	TAS [21,23]
		Genus *Treponema*	TAS [21,24,25]
		Species *Treponema succinifaciens*	TAS [1,2]
		Type strain 6091	TAS [1,2]
	Gram stain	negative	TAS [1]
	Cell shape	helical-shaped	TAS [1]
	Motility	motile	TAS [1]
	Sporulation	none	TAS [1]
	Temperature range	22–43°C	TAS [1]
	Optimum temperature	35–39°C	TAS [1]
	Salinity	not reported	
MIGS-22	Oxygen requirement	anaerobic	TAS [1]
	Carbon source	carbohydrates	TAS [1]
	Energy metabolism	chemoorganotroph	TAS [1]
MIGS-6	Habitat	intestine of healthy pigs	TAS [1]
MIGS-15	Biotic relationship	free-living	NAS
MIGS-14	Pathogenicity	none	NAS
	Biosafety level	1	TAS [26]
	Isolation	colon of swine	TAS [1]
MIGS-4	Geographic location	USA	TAS [1]
MIGS-5	Sample collection time	1972 or before	TAS [1]
MIGS-4.1	Latitude	not reported	
MIGS-4.2	Longitude	not reported	
MIGS-4.3	Depth	not reported	
MIGS-4.4	Altitude	not reported	

Evidence codes - IDA: Inferred from Direct Assay (first time in publication); TAS: Traceable Author Statement (i.e., a direct report exists in the literature); NAS: Non-traceable Author Statement (i.e., not directly observed for the living, isolated sample, but based on a generally accepted property for the species, or anecdotal evidence). These evidence codes are from of the Gene Ontology project [27]. If the evidence code is IDA, the property was directly observed by one of the authors or an expert mentioned in the acknowledgements.

### Chemotaxonomy

No chemotaxonomic information is currently available for *T. succinifaciens*.

## Genome sequencing and annotation

### Genome project history

This organism was selected for sequencing on the basis of its phylogenetic position [30], and is part of the ***G****enomic* ***E****ncyclopedia of* ***B****acteria and* ***A****rchaea* project [31]. The genome project is deposited in the Genomes On Line Database [15] and the complete genome sequence is deposited in GenBank. Sequencing, finishing and annotation were performed by the DOE Joint Genome Institute (JGI). A summary of the project information is shown in Table 2.

**Table 2:** Genome sequencing project information

**MIGS ID**	**Property**	**Term**
MIGS-31	Finishing quality	finished
MIGS-28	Libraries used	Three genomic libraries: one 454 pyrosequence standard library, one 454 PE library (10.5 kb insert size), one Illumina library
MIGS-29	Sequencing platforms	Illumina GAii, 454 GS FLX Titanium
MIGS-31.2	Sequencing coverage	960.0 × Illumina; 47.3 × pyrosequence
MIGS-30	Assemblers	Newbler version 2.3, Velvet version 0.7.63, phrap version SPS - 4.24
MIGS-32	Gene calling method	Prodigal 1.4, GenePRIMP
	INSDC ID	CP002631
	Genbank Date of Release	April 15, 2011
	GOLD ID	Gc01722
	NCBI project ID	50825
	Database: IMG-GEBA	2504557012
MIGS-13	Source material identifier	DSM 2489
	Project relevance	Tree of Life, GEBA

### Growth conditions and DNA isolation

*T. succinifaciens* strain 6091^T^, DSM 2489, was grown anaerobically in DSMZ medium 275 (*Treponema succinifaciens* medium) [32] at 37°C. DNA was isolated from 0.5-1 g of cell paste using MasterPure Gram-positive DNA purification kit (Epicentre MGP04100) following the standard protocol as recommended by the manufacturer with modification st/DL for cell lysis as described in Wu *et al*. 2009 [31]. DNA is available through the DNA Bank Network [33].

### Genome sequencing and assembly

The genome was sequenced using a combination of Illumina and 454 sequencing platforms. All general aspects of library construction and sequencing can be found at the JGI website [34]. Pyrosequencing reads were assembled using the Newbler assembler (Roche). The initial Newbler assembly consisting of 134 contigs in two scaffolds was converted into a phrap assembly [35] by making fake reads from the consensus, to collect the read pairs in the 454 paired end library. Illumina sequencing data (3,531 Mb) was assembled with Velvet [36] and the consensus sequences were shredded into 1.5 kb overlapped fake reads and assembled together with the 454 data. The 454 draft assembly was based on 136.1 Mb 454 draft data and all of the 454 paired end data. Newbler parameters are -consed -a 50 -l 350 -g -m -ml 20. The Phred/Phrap/Consed software package [35] was used for sequence assembly and quality assessment in the subsequent finishing process. After the shotgun stage, reads were assembled with parallel phrap (High Performance Software, LLC). Possible mis-assemblies were corrected using gapResolution [34], Dupfinisher [37] or sequencing cloned bridging PCR fragments with subcloning. Gaps between contigs were closed by editing in Consed [35], by PCR and by Bubble PCR primer walks (J.-F. Chang, unpublished). A total of 305 additional reactions were necessary to close gaps and to raise the quality of the finished sequence. Illumina reads were also used to correct potential base errors and increase consensus quality using a software Polisher developed at JGI [38]. The error rate of the completed genome sequence is less than 1 in 100,000. Together, the combination of the Illumina and 454 sequencing platforms provided 1,007.3 × coverage of the genome. The final assembly contained 486,725 pyrosequence and 36,577,056 Illumina reads.

### Genome annotation

Genes were identified using Prodigal [39] as part of the Oak Ridge National Laboratory genome annotation pipeline, followed by a round of manual curation using the JGI GenePRIMP pipeline [40]. The predicted CDSs were translated and used to search the National Center for Biotechnology Information (NCBI) non-redundant database, UniProt, TIGR-Fam, Pfam, PRIAM, KEGG, COG, and InterPro databases. Additional gene prediction analysis and functional annotation was performed within the Integrated Microbial Genomes - Expert Review (IMG-ER) platform [41].

## Genome properties

**Figure 3:**
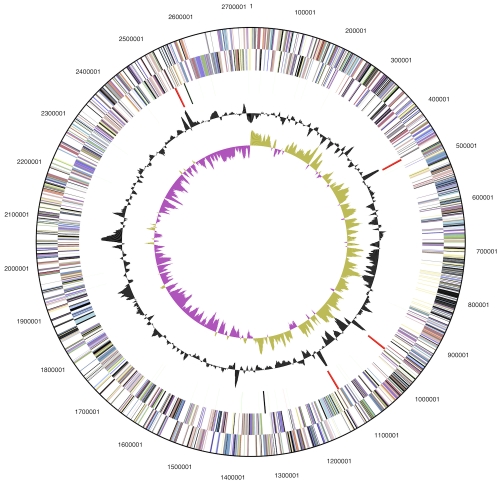
Graphical map of the chromosome (not drawn to scale with plasmid in Figure 4). From bottom to top: Genes on forward strand (color by COG categories), Genes on reverse strand (color by COG categories), RNA genes (tRNAs green, rRNAs red, other RNAs black), GC content, GC skew.

**Figure 4:**
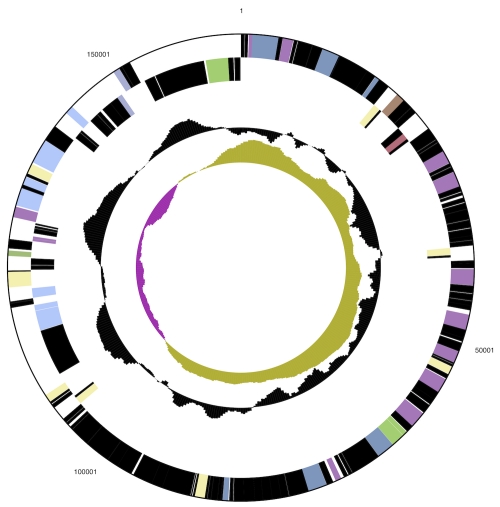
Graphical map of the plasmid  (not drawn to scale with chromosome in Figure 3). From bottom to top: Genes on forward strand (color by COG categories), Genes on reverse strand (color by COG categories), RNA genes (tRNAs green, rRNAs red, other RNAs black), GC content, GC skew.

The genome consists of a 2,731,853 bp long chromosome and a 165.572 bp long plasmid both with a G+C content of 39.1% (Table 3, Figure 3and Figure 4). Of the 2,786 genes predicted, 2,723 were protein-coding genes, and 63 RNAs; 115 pseudogenes were also identified. The majority of the protein-coding genes (57.8%) were assigned a putative function while the remaining ones were annotated as hypothetical proteins. The distribution of genes into COGs functional categories is presented in Table 4.

**Table 3:** Genome Statistics

**Attribute**	**Value**	**% of Total**
Genome size (bp)	2,897,425	100.00%
DNA coding region (bp)	2,550,315	88.02%
DNA G+C content (bp)	1,133,894	39.13%
Number of replicons	2	
Extrachromosomal elements	0	
Total genes	2,786	100.00%
RNA genes	63	2.26%
rRNA operons	4	
Protein-coding genes	2,723	97.74%
Pseudo genes	115	4.13%
Genes with function prediction	1,611	57.82%
Genes in paralog clusters	373	13.39%
Genes assigned to COGs	1,674	60.09%
Genes assigned Pfam domains	1,800	64.61%
Genes with signal peptides	812	29.15%
Genes with transmembrane helices	581	20.85%
CRISPR repeats	1	

**Table 4:** Number of genes associated with the general COG functional categories

**Code**	**value**	**%age**	**Description**
J	142	7.8	Translation, ribosomal structure and biogenesis
A	0	0.0	RNA processing and modification
K	120	6.6	Transcription
L	179	9.8	Replication, recombination and repair
B	0	0.0	Chromatin structure and dynamics
D	24	1.3	Cell cycle control, cell division, chromosome partitioning
Y	0	0.0	Nuclear structure
V	46	2.5	Defense mechanisms
T	103	5.7	Signal transduction mechanisms
M	120	6.6	Cell wall/membrane/envelope biogenesis
N	63	3.5	Cell motility
Z	1	0.0	Cytoskeleton
W	0	0.0	Extracellular structures
U	61	3.3	Intracellular trafficking, secretion, and vesicular transport
O	55	3.0	Posttranslational modification, protein turnover, chaperones
C	74	4.1	Energy production and conversion
G	111	6.1	Carbohydrate transport and metabolism
E	128	7.0	Amino acid transport and metabolism
F	62	3.4	Nucleotide transport and metabolism
H	58	3.2	Coenzyme transport and metabolism
I	34	1.9	Lipid transport and metabolism
P	60	3.3	Inorganic ion transport and metabolism
Q	5	0.3	Secondary metabolites biosynthesis, transport and catabolism
R	240	13.2	General function prediction only
S	138	7.6	Function unknown
-	1,112	39.9	Not in COGs
